# Non-Intubated Thoracic Surgery: Standpoints and Perspectives

**DOI:** 10.3389/fsurg.2022.937633

**Published:** 2022-07-01

**Authors:** Marco Anile, Jacopo Vannucci, Francesco Ferrante, Katia Bruno, Dalila De Paolo, Massimiliano Bassi, Francesco Pugliese, Federico Venuta, the NIVATS Interest Group

**Affiliations:** ^1^ Department of Thoracic Surgery and Lung Transplantation, University of Rome Sapienza, Policlinico Umberto I, Rome, Italy; ^2^ Department of Anesthesiology and Critical Care, University of Rome Sapienza, Policlinico Umberto I, Rome, Italy

**Keywords:** Non-intubated surgery, video-assisted thoracic surgery, NI-VATS, pulmonary resections, perioperative management, anesthesia

## Abstract

Non-intubated video-assisted thoracic surgery (NI-VATS) combines the advantages of a non-intubated surgery with the benefits of a minimally invasive approach. First, NI-VATS is performed in the case of fragile patients when general anesthesia and/or orotracheal intubation can be foreseen as inconvenient. However, NI-VATS indications have been increasingly extended to different patient conditions, considering the increasingly assessed safety and feasibility of the procedure. Currently, the NI-VATS approach is used worldwide for different thoracic surgery procedures, including the management of malignant pleural effusion, surgical treatment of empyema, anatomical and non-anatomical lung resection, and other indications. In fact, this approach has shown to be less impactful than VATS under general anesthesia, allowing for shortened hospitalization and faster recovery after surgery. Besides, NI-VATS is associated with fewer pulmonary complications, less respiratory distress, and a mild systemic inflammatory reaction. For these reasons, this approach should be considered not only in patients with poor cardiac or respiratory function (general functional reserve), but also in other eligible conditions.

We explored the anesthetic and surgical aspects of such an approach, including the management of analgesia, cough reflex, depth of sedation, and intraoperative technical issues to put this approach in perspective.

## Introduction

Non-intubated thoracic surgery (NITS) is a particular approach that has been developed during the last two decades (1). NITS is defined by a patient's spontaneous breathing during the entire operation and avoidance of neuromuscular block (2). The purpose of this technique is to minimize the impact of anesthesia on patients undergoing surgery. During such a procedure, lung ventilation is not managed by orotracheal intubation at all. Besides, the use of supraglottic devices (e.g. laryngeal mask, high flow nasal cannula) is differently employed to support patient breathing with several possible approaches.

This anesthesiologic method is sometimes applied to Video Assisted Thoracic Surgery (VATS) with the scope of reducing the general operative stress on patients with increasing results and promising perspectives. NITS is usually hired, even if not completely reserved, when a minimally invasive procedure is planned, and has created a new subset of VATS: the NI-VATS (Nonintubated-Video Assisted Thoracic Surgery). NI-VATS represents the vast majority of non-intubated techniques, but there are some reports of NITS in other operative conditions. Al-Abdullatief et al. performed thoracotomy and sternotomy under spontaneous breathing (3). Notwithstanding the possibility for a successful use of NITS in impactful surgery, this is anecdotal.

NI-VATS has been traditionally considered to be an appropriate technique in the case of fragile patients with compromised cardiopulmonary function (4, 5). One of the earliest successful non-intubated VATS was performed in 1998 by Mukaida et al. (5) in high-risk patients. In this series, four patients with secondary pneumothorax were submitted to surgery under local or epidural anesthesia. The authors could observe a good outcome and proposed to investigate more deeply the potential role of spontaneous breathing to preserve patients from mechanical ventilation damage.

Patients with chronic obstructive pulmonary disease (COPD) and other comorbidities can experience reactive events such as bronchospasm or even worse pulmonary impairment in the perioperative period after intubation; therefore, NI-VATS seems to represent a valid alternative to traditional management for different reasons. One lung ventilation (OLV) in lateral decubitus, with the dependent lung below, is the standard condition in traditional VATS; this approach is associated with possible complications in marginal patients for chronic cardiorespiratory disease (6). At the same time, NITS has elements of weakness. Indeed, lateral decubitus and iatrogenic pneumothorax after opening the pleural cavity lead to remarkable changes in the Ventilation/Perfusion ratio (V/Q), sometimes triggering respiratory impairment, mostly transient but potentially threatening. In this regard, in non-intubated procedures, some differences in ventilation dynamics compared with mechanical ventilation pendulum are described because of spontaneous breathing and the lack of OLV (7). First of all, carbon dioxide rebreathing occurs; inspirated air volume in the dependent lung is partially exhaled into the non-dependent lung during the expiration phase and re-inspirated by the dependent lung once again in the following phase. This pendulum-like movement could lead to hypoxia and hypercapnia. This occurrence represents one of the most frequent reasons for conversion to orotracheal intubation. Moreover, although hypercapnia could represent an issue for non-intubated surgery, there is quite a bit of tolerance for this condition. In fact, the so called permissive hypercapnia is expected to improve V/Q through hypoxic pulmonary vasoconstriction (HPV), increasing parenchymal compliance and small airway dilation directly (8).

Over the years, the role of NI-VATS has changed. In the earliest experience, NI-VATS was a dedicated approach to patients in whom general anesthesia and orotracheal intubation were thought to be harmful, even if no absolute contraindications to general anesthesia were assessed. More recently, NI-VATS has largely been applied more to the conditions of different patients. Because of the reported safety and mild impact, NI-VATS can represent a reliable option for several thoracic procedures (8, 9). Gonzalez-Rivas et al. reported that limited pulmonary resections can be performed through awake or minimally sedated patients (10). Promptly, NI-VATS has been explored for more advanced procedures. Anatomic lung resections, and even the most challenging ones, have been performed without intubation. In addition to major lung resections, management of malignant pleural effusion, surgical treatment of empyema, bronchial sleeve procedures, and tracheal resections were described using a non-intubated approach (10) also with robotic assistance (11). These reports are still to be considered initial experiences, and procedures are performed in highly dedicated centers and not yet represent the standard of care.

## Contraindications to the Procedure

Patients with known or suspected difficult intubation (Mallampati III–IV), body mass index >30 kg/m^2^, persistent cough, basal *p*O2 < 60 mmHg and basal *p*CO2 > 50 mmHg, coagulopathy (International Normalized Ratio >1.5), and contralateral phrenic nerve palsy are not eligible for this approach (10). Obese patients are excluded from NI-VATS indication because of wide respiratory movement that adipose tissue can provoke. The technical condition in obese patients is hampered; technically difficult procedures warrant a strong recommendation to avoid non-intubated procedures when patient-related issues are assessed. Besides, a difficult intubation is a solid contraindication for the procedure because conversion to orotracheal intubation must be foreseen in every case, and to perform a rapid intubation in lateral decubitus is a challenging task. Expected pleuroparenchymal adhesions, previous thoracic surgery, neoadjuvant chemotherapy and/or radiotherapy, and central masses are relative contraindications to this approach: in these cases, non-intubated surgery can be performed, but an experienced team and expert hands seem to be crucial for a positive outcome (10, 12). When the above-mentioned conditions are present, the conversion rate dramatically increases.

## Anesthetic Considerations

General anesthesia and orotracheal intubation, with subsequent mechanical ventilation, has several disadvantages:

∘  ventilator-induced lung injury (VILI); mechanical ventilation can result in interstitial edema, surfactant loss, decreased compliance, V/Q impairment (13), and release of inflammation mediators (10, 14). In a non-dependent lung, a hypoxic environment is created, which causes acidosis, alveolar edema, vascular congestion, and the release of pro-inflammatory cytokines (15). These changes can occur even without surgical trauma. During re-expansion of the non-dependent lung, the use of high FiO_2_ causes oxidative stress (16) with neutrophil chemotaxis and macrophage activation; this inflammatory activation increases the risk of arrhythmias and QT elongation (17, 18) and can also be detected through the measurement of C-reactive protein levels (19);
∘  the use of volatile anesthetics has a more significant impact on HPV compared with sedation obtained with Propofol;
∘  atelectasis of some areas of the dependent lung, also due to a paralysis of the ipsilateral hemidiaphragm; it is followed by a worsening of the V/Q, already limited by the lateral decubitus and by the one lung ventilation (OLV);
∘  lesions of the airways and the vocal cords consequent to intubation; this event is infrequent, approximately 1 case in 20000 intubations (20), but the mortality rate in tracheobronchial ruptures is approximately 22% (12);
∘  post-operative pharyngodynia, due to intubation maneuvers;
∘  residual neuromuscular blockade in the post-operative period (21).

Moreover, following the reported disadvantages, NI-VATS showed a lower impact on lymphocyte activation (22) and stress hormone response (23) compared with traditional VATS with orotracheal intubation. In a randomized control trial, Liu et al. (7) reported a shorter hospital stay, less post-operative complications, and earlier oral intake resume in patients submitted to the NI-VATS approach compared with those undergoing VATS with general anesthesia. Deng et al. achieved the same conclusions in their meta-analysis (24).

## Management of Analgesia

When general anesthesia is not scheduled, the procedure requires different strategies to obtain sedation and analgesia. The most commonly used techniques to obtain analgesia are neuraxial blockade like thoracic epidural anesthesia (TEA), paravertebral blockade (PVB), regional anesthesia like erector spinae plane block (ESPB), and serratus anterior blockade (25). These techniques are used to support analgesia also after general anesthesia.

TEA is a reliable and frequently used technique; it provides bilateral block, but it is associated with possible complications like hypotension, epidural hematomas, nerve injury, and urinary retention (12). PVB has a unilateral effect, prevents epidural hematomas, and can also be performed in case of spine deformity and vertebral abnormalities when TEA is unlikely to succeed. ESPB was initially used for chronic pain after thoracotomy and more recently adopted to perioperatively manage pain after VATS. Its use seems promising in the context of NI-VATS. ESPB is often performed with ultrasound guidance and has a unilateral effect, like PVB. It appears safer than TEA, because it does not entail the risk of nerve injury. Furthermore, analgesia is usually supported with opioids like Fentanyl and Remifentanil: the latter has an ultrashort half-life, so its effect on ventilation is predictable and easily balanced to prevent hypoxia and apnea.

## Cough Reflex

A key issue in NI-VATS is the need to minimize the cough reflex. It is provoked by the manipulation of the lung parenchyma, especially near bronchi, and can represent a consistent obstacle to the procedure (7). In some cases, the limitation caused by cough represents a reason to convert to general anesthesia and intubation (26). Locoregional anesthesia does not suppress visceral pleural sensitivity, and, therefore, blockade of pleural sensitivity is required to avoid cough reflex and needs to be well accomplished. It is performed by means of several methods:

– infiltration of local anesthetic (lidocaine or bupivacaine) near the vagus nerve (26–28) during VATS; Liu et al. recommend identifying the vagus nerve between trachea and superior vena cava in right-sided procedures and in the aortopulmonary window in left-sided procedures (7);
– inhalation of aerosolized lidocaine just before the procedure (6, 12);
– spray instillation of lidocaine on the lung surface (12);
– stellate ganglion block (12).

In our experience, inhalation of 5 ml of aerosolized lidocaine 2% for about 20 minutes before surgery with a flow rate of 10 L/min is effective in preventing cough reflex during wedge pulmonary resections or pleural procedures. Considering the available data in the current literature, vagus nerve infiltration is probably more effective when compared with the other described technique, and its use is advocated in major thoracic procedures such as pulmonary lobectomies (8).

## Sedation and Anesthesia Depth

Non-intubated thoracic surgery can be conducted with various grades of sedation, from complete consciousness in which patients can interact with surgeons, to deep sedation that precludes verbal response but allows spontaneous breathing (28). In 2008, Pompeo et al. reported a series of fully awake NI-VATS pulmonary resections (29). The main issue related to this state of consciousness is the risk of developing panic attacks, which can be managed conservatively by interacting with the patient during the procedure if the event is mild or by increasing the depth of anesthesia in case of more severe events. In the NI-VATS context, because of the lack of tracheal intubation, sedation can be obtained only via Propofol infusion, thus performing a total intravenous anesthesia (TIVA).

Depth of sedation is an important parameter for NI-VATS; it can be measured through the Bi-spectral (BIS) index. BIS index monitoring generates a numerical value: a number between 90 and 60 is indicative of a state of sedation. If higher than 90, it indicates the awake state, while a value below 60 stands for deep sedation (28). Sedation and deep sedation status allow surgical maneuvers more easily than awake status, but Propofol level control is strictly required to maintain spontaneous breathing and airway reflex. In this condition, oxygen administration through nasal cannula, laryngeal mask, or facial mask is recommended (10).

## Surgical Concerns

Several intrinsic characteristics of spontaneous breathing surgery can cause difficulties to the surgical procedure. The main issue related to this approach is the movement of the diaphragm and the mediastinal shifting; these aspects are relevant both for the surgeon and for the assistant because several surgical paradigms change. The assistant needs to adapt the action to the respiratory movement to keep the surgical field in frame and in a condition of good exposure. The surgeon must consider the possible repetitive need to temporarily interrupt the procedure during coughing or muscle contraction. If cough, mediastinal movement, or unbalanced respiration are recurring, the procedure tends to conversion. Chiang et al. provided a review of the literature about the reasons for conversion in non-intubated thoracoscopic surgery (26). They could identify several important circumstances: severe adhesions, major bleeding, and serious mediastinal and diaphragmatic movements are the most frequent surgical issues in this approach. Moreover, considering that the lungs are not taken apart from each other (no cuffed bilumen orotracheal tube nor an endobronchial blocker), a non-dependent lung could be affected by paradoxical breathing, the lung collapses during inspiration, and it expands during exhalation. All these events could represent limiting factors for a successful procedure. They are recognized to be technical hurdles, but they have not yet been reported to be absolute indications for conversion.

## Anesthesiologic Issues

A crucial aspect of spontaneous breathing anesthesia is the trending increase of *p*CO_2_ during the surgical procedure. Regarding contraindications and indication to conversion to oro-tracheal intubation, there are several different positions. Generally, an excessive *p*CO_2_ increase (over 80 mmHg) is one of the most frequent causes of conversion to intubation. Other common reasons to conversion are *p*H < 7,1, *p*O2 < 60 mmHg, and persistent cough during surgery (10). The rate of conversion in the literature ranges between 2% and 12% (26); team experience might have an impact on the chosen parameters’ range of tolerance to conversion or patient selection. A wide spectrum of safety using a stricter parameter range could lead to early conversion or to patient exclusion from NIVATS, which is not a bad choice if the team is inexperienced or not confident.

## Exemplifying Case

We present the case of a 43-year-old women with a history of breast cancer undergoing surgery and adjuvant chemo-radiotherapy. During the follow-up, she was diagnosed with a pulmonary nodule in the right middle lobe. She had no other comorbidity and a normal cardiac function (Left ventricle ejection fraction = 59%, Pulmonary artery systolic pressure (PASP)  =  36mmHg estimated through transthoracic echocardiography) and preoperative arterial gas examination showed *p*CO_2 _= 35 mmHg and *p*O_2 _= 93 mmHg.

**Figure 1:**
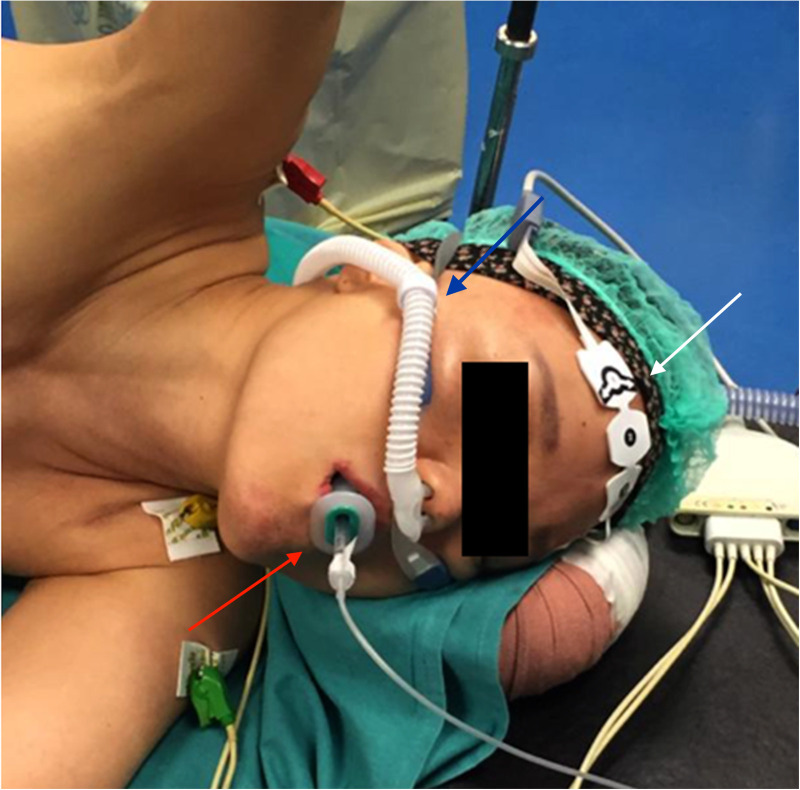
Anesthetic setting. Capnograph was passed through the Guedel airway device (red arrow). Spontaneous breathing was supported by high flow nasal cannula (blue arrow), and the depth of anesthesia was monitored using the bi-spectral index (white arrow).

The patient was evaluated for surgery and NI-VATS was scheduled. She was supported with High Flow Nasal Cannula (HFNC) during surgery; the depth of anesthesia was monitored with the BIS index, and the maintenance of spontaneous breathing was assessed through capnometry. Capnograph was passed through the Guedel airway device, only to verify the maintenance of breathing and not to evaluate EtCO_2_ (Figure 1). The BIS index was maintained in the range of deep sedation (lower than 60). Electrocardiographic activity was monitored continuously, and invasive blood pressure and peripheral hemoglobin saturation were registered. Regional analgesia was obtained through the ESBP block (150 mg of Ropivacaine and 4 mg of Dexamethasone are injected through a 22-gauge needle at T5 level). Twenty minutes before skin incision, lidocaine was administered via aerosol to suppress cough reflex.

Anesthesia was induced with 2 mg of Midazolam, Propofol, and Remifentanil and maintained via continuous infusion of Remifentanil (0.05–1 μg/kg/min) and Propofol (2–4 mg/kg/h, titrated during the procedure according to the BIS value), thus performing TIVA.

The patient was submitted to a uniportal VATS right middle lobe wedge resection. Extended pleural and pericardial adhesions were dissected, and the nodule was targeted and removed by staplers. The operative time was 80 min. Blood gas values remained within the normal range, so the procedure was carried out in spontaneous breathing and through the uniportal VATS approach. The excellent preoperative condition, young age, and the good functional reserve supported an ideal operative condition. Fluid intake per os was resumed after 4 h, the patient could walk after 6 h, and food oral intake was started after 8 h. Chest drainage was removed on post-operative day 1, and the patient was discharged on post-operative day 2, who had no complaints of symptoms and pain.

## Perspectives

The NIVATS technique was first used to support marginal patients and then was adopted to fit patients with even better results. According to this observation, over the years, a wider dissemination of NIVATS in low-impact procedures seems to be clearly indicated in a very large population, ranging from severely affected patients to younger and fit ones. In fact, non-intubated surgery has also proven its feasibility in major procedures, but outlining concrete perspectives is quite impossible due to the lack of evidence and technical issues. However, NIVATS provides excellent opportunities for a speedy recovery in minor procedures and could become the standard of choice for such operations.

With regard to patient management, oxygenation support is frequently needed. The most common devices are Venturi mask, facial mask, nasal cannula, and laryngeal mask (10). In our experience, we adopted HFNC, and oxygen flow is continuously adjusted to maintain peripheral oxygen saturation >95%. Currently, new opportunities arise from technological improvements in oxygen delivery from mechanical ventilators that can host HFNC.

## Conclusions

After initial hesitation, NI-VATS has shown improvement. Currently, there are several reports that encourage dedicated surgeons and scientists to explore more systematically the role of NI-VATS even if these reports are few and sparse. NI-VATS is a safe and feasible approach that can be proposed for minor and major thoracic procedures. With regard to major procedures, data are insufficient to come to definitive conclusions, but with regard to minor procedures, NI-VATS has shown to be less impactful than VATS performed under general anesthesia. NI-VATS facilitates shortened hospital stay and promotes speedy recovery after surgery. For these reasons, it can be offered not only to patients with poor cardiac or respiratory function, but also to patients with other eligible conditions with good potential.

## Data Availability

The original contributions presented in the study are included in the article/Supplementary Material, and further inquiries can be directed to the corresponding author/s.
